# A longitudinal PET study on changes in brain norepinephrine transporter availability following duloxetine treatment in major depressive disorder

**DOI:** 10.1093/ijnp/pyaf064

**Published:** 2025-09-11

**Authors:** Sho Moriguchi, Keisuke Takahata, Harumasa Takano, Hironobu Endo, Kenji Tagai, Soichiro Kitamura, Hiroyuki Uchida, Masaru Mimura, Manabu Kubota, Ming-Rong Zhang, Makoto Higuchi

**Affiliations:** Advanced Neuroimaging Center, Institute for Quantum Medical Science, National Institutes for Quantum Science and Technology (QST), Chiba, Japan; Department of Neuropsychiatry, Keio University School of Medicine, Tokyo, Japan; Advanced Neuroimaging Center, Institute for Quantum Medical Science, National Institutes for Quantum Science and Technology (QST), Chiba, Japan; Department of Neuropsychiatry, Keio University School of Medicine, Tokyo, Japan; Department of Psychiatry, National Center of Neurology and Psychiatry, Tokyo, Japan; Advanced Neuroimaging Center, Institute for Quantum Medical Science, National Institutes for Quantum Science and Technology (QST), Chiba, Japan; Advanced Neuroimaging Center, Institute for Quantum Medical Science, National Institutes for Quantum Science and Technology (QST), Chiba, Japan; Advanced Neuroimaging Center, Institute for Quantum Medical Science, National Institutes for Quantum Science and Technology (QST), Chiba, Japan; Department of Neuropsychiatry, Keio University School of Medicine, Tokyo, Japan; Department of Neuropsychiatry, Keio University School of Medicine, Tokyo, Japan; Department of Neuropsychiatry, Kyoto University, Kyoto, Japan; Department of Advanced Nuclear Medicine Sciences, Institute for Quantum Medical Science, National Institutes for Quantum Science and Technology (QST), Chiba, Japan; Advanced Neuroimaging Center, Institute for Quantum Medical Science, National Institutes for Quantum Science and Technology (QST), Chiba, Japan

**Keywords:** major depressive disorder, norepinephrine transporter, PET analysis, duloxetine, antidepressant

## Abstract

**Importance:**

The norepinephrine transporter (NET) plays a crucial role in major depressive disorder (MDD). Serotonin and norepinephrine reuptake inhibitors, such as duloxetine, are first-line treatments for MDD. Duloxetine inhibits the reuptake of both serotonin and norepinephrine. However, its precise mechanism, particularly regarding NET occupancy and changes in transporter availability, remains unclear. Norepinephrine transmission, mediated by NET, may be instrumental in treating MDD. Therefore, NET occupancy could serve as a potential biomarker to evaluate treatment efficacy.

**Objective:**

This study evaluated duloxetine’s impact on brain NET availability in patients with MDD using positron emission tomography (PET) with (*S,S*)-[^18^F]FMeNER-D_2_ and its correlation with clinical symptoms using the Hamilton Depression Rating Scale (HAM-D).

**Design:**

Longitudinal study.

**Setting:**

Psychiatric hospitals and clinics.

**Participants:**

Fifteen patients with MDD.

**Interventions:**

Duloxetine (20–60 mg daily).

**Main Outcomes and Measures:**

Baseline PET examinations were conducted for all patients, followed by treatment with duloxetine (20–60 mg daily). After 4–6 weeks of duloxetine administration, a second PET examination was performed, and plasma concentrations of duloxetine were measured immediately before and after the second examination using gas chromatography–mass spectrometry. Seven patients showing symptom improvement over the course of time discontinued duloxetine and underwent a third PET examination after a 2-month washout period.

**Results:**

Norepinephrine transporter occupancy by duloxetine was 30%–40% across the doses studied. A paired *t*-test comparing NET availability before and after duloxetine treatment showed no significant differences, suggesting that duloxetine did not significantly alter NET availability in the short term. Further analysis revealed a significant positive correlation between the change in NET availability and HAM-D scores after treatment. Patients with greater reductions in HAM-D scores exhibited more pronounced reductions in NET availability.

**Conclusions:**

These findings underscore the potential role of NET occupancy by duloxetine in the treatment of MDD and its relationship with clinical outcomes.

**Relevance:**

Understanding changes in NET and their implications enhances our comprehension of the complex mechanisms behind antidepressants and may reveal new therapeutic targets for MDD and other neuropsychiatric disorders.

**Clinical trial registration number:**

UMIN000008251.

Significance Statement This study investigated how the antidepressant duloxetine influences the norepinephrine transporter (NET) occupancy in the brains of individuals with major depression. We measured NET occupancy in 15 patients before and after treatment using positron emission tomography (PET). Initially, participants underwent a PET examination, followed by 4–6 weeks of duloxetine administration and a second examination. Those who showed symptom improvement over the course of time discontinued the medication and received a final examination 2 months later. Duloxetine produced measurable NET occupancy (~30–40%), and the magnitude of change in NET availability was associated with clinical severity after treatment. This is the first study to monitor such long-term effects using a PET examination without the influence of additional medications. Our findings emphasize the role of norepinephrine transmission in treating major depressive disorder and suggest that NET occupancy may serve as a promising biomarker for treatment effectiveness.

## INTRODUCTION

Major depressive disorder (MDD) is a prevalent psychiatric disorder arising from a complex interplay of social, psychological, and physiological factors. Due to its high prevalence, recurring episodes, and significant psychosomatic impact, MDD imposes a considerable social and economic burden. It is the leading cause of disability in developed countries and the fourth leading cause of disability worldwide.^1^ Major depressive disorder is characterized by affective, cognitive, and somatic symptoms such as persistent low mood, loss of interest, cognitive impairments, and physical discomfort. Multiple pathologies are implicated in MDD, including the dysregulation of monoamine neurotransmitters, such as serotonin, norepinephrine, and dopamine.^2^ The critical role of norepinephrine in neural systems contributing to MDD symptoms has long been recognized.^3^ However, findings from postmortem studies on norepinephrine transporter (NET) availability in MDD have yielded mixed and inconclusive results. Some studies indicate reduced NET binding in the locus coeruleus of patients with MDD, which may reflect a compensatory downregulation of NET expression in response to chronically reduced norepinephrine release, suggesting a decline in overall noradrenergic activity.^4^ Other findings show different alterations in adrenergic receptors, such as increased beta-adrenergic receptor binding in the cortex of suicide victims^5^ and higher alpha2- and beta1-adrenoceptor densities in the frontal cortex of antidepressant-free patients with MDD.^6^^,^^7^ These varying outcomes underscore the complexity of norepinephrine system alterations in MDD and the need for further in vivo studies to clarify these findings. In addition, genetic factors play a role in MDD, with evidence of reduced gene expression in locus coeruleus astrocytes in men with MDD,^8^ potentially contributing to disrupted norepinephrine and glutamate transmission. In contrast, several in vivo studies have provided clearer insights into the connection between the norepinephrine system and MDD. One prominent study by Moriguchi et al.^3^ using positron emission tomography (PET) with (*S,S*)-[^18^F]FMeNER-D_2_ showed that patients with MDD have significantly higher NET binding potential in the thalamus compared to healthy controls. This suggests that elevated NET in regions such as the thalamus may enhance norepinephrine reuptake, contributing to reduced synaptic norepinephrine transmission in MDD. Serotonin and norepinephrine reuptake inhibitors (SNRIs) may counteract this by inhibiting NET, thereby restoring norepinephrine signaling and alleviating depressive symptoms.

Previous research has shown that norepinephrine pathways regulate cognitive functions such as attention, memory, mood, motivation, and arousal.^9^ The NET, located on the presynaptic membrane of noradrenergic neurons, is critical in terminating norepinephrine’s action by transporting it back into the presynaptic neuron.^10^ This makes NET a key target for various antidepressants, including SNRIs, which increase norepinephrine concentration in the synaptic cleft, thereby enhancing noradrenergic neurotransmission and relieving depressive symptoms. Serotonin and norepinephrine reuptake inhibitors, such as duloxetine, are commonly prescribed as first-line treatments for MDD,^11^ with the dose determined based on serotonin transporter (SERT) occupancy.^12^^,^^13^ Duloxetine functions by inhibiting the reuptake of both serotonin and norepinephrine, combining 2 therapeutic mechanisms to effectively treat depression and anxiety. The underlying mechanisms of SNRIs are complex because they act on both serotonin and norepinephrine; therefore, further research is warranted to elucidate their actual mode of action in various pathologies.

Despite their proven clinical effectiveness, the precise mechanisms of SNRIs, particularly concerning NET occupancy and changes in transporter availability, remain unclear. Therefore, this study was conducted to evaluate the impact of duloxetine treatment on brain NET availability in patients with MDD using PET with (*S,S*)-[^18^F]FMeNER-D_2_. Specifically, the study aimed to measure NET occupancy in 15 patients with MDD before and after duloxetine administration (20–60 mg) for 4–6 weeks, and investigate the relationship between changes in NET availability and clinical outcomes, as measured by the Hamilton Depression Rating Scale (HAM-D). In addition, NET availability was assessed after tapering off duloxetine and a 2-month wash out period to elucidate the long-term effects of duloxetine treatment. This study aimed to enhance understanding of how duloxetine impacts NET in the brain and its relevance for treating MDD. By elucidating the changes in NET availability and their correlation with clinical symptoms, this study seeks to offer insights into the mechanisms of action of SNRIs and guide future therapeutic strategies for MDD.

## METHODS

### Participants

This longitudinal study included 15 patients (mean age = 39.7 years, standard deviation [SD] = 10.3, range = 20–54 years) meeting DSM-IV criteria for MDD, recruited from 2 affiliated psychiatric hospitals and 2 clinics in Chiba, Japan between October 1, 2012 and March 31, 2016.

All participants had no history of major medical illnesses. Lifetime psychiatric disorders were evaluated using the Mini-International Neuropsychiatric Interview,^14^ and patients were determined to be free from comorbid psychiatric disorders. None of the patients had received psychopharmacologic treatment for at least 1 year before the study. Symptom severity was measured using the Japanese version of the 17-item HAM-D scale^15^ on the day of the PET examinations. The relationship between duloxetine dose, resulting NET occupancy, and changes in clinical symptoms was listed as the primary outcome at the time of trial registration.

### Ethics Statement

The authors assert that all procedures contributing to this work comply with the ethical standards of the relevant national and institutional committees on human experimentation and with the Helsinki Declaration of 1975, as revised in 2013. All procedures involving human subjects were approved by the Radiation Drug Safety Committee and the Institutional Review Board of the National Institute of Radiological Sciences (approval number: 11-023). The study was registered on June 25, 2012, at the University Hospital Medical Information Network Clinical Trials Registry (registration number: UMIN000008251) before initiating the enrolment of participants.

### Consent Statement

All participants provided written informed consent for participation in the study.

### PET and MRI Acquisition

Positron emission tomography examinations were performed using an ECAT EXACT HR+ scanner (CTI-Siemens, USA) with a 15.5-cm axial field of view. A 10-min transmission scan for attenuation correction was conducted using a ^68^Ge-^68^Ga source. The radioligand (*S,S*)-[^18^F]FMeNER-D_2_ was synthesized by fluoromethylation of norethylreboxetine with ^18^F-bromofluoro-methane-d_2_^16^ with over 95% radiochemical purity. Emission data were acquired between 120 and 180 min post-injection (10 min × 6 frames) in 3-dimensional mode after an intravenous bolus of (*S,S*)-[^18^F]FMeNER-D_2_. Based on prior modelling studies with (*S,S*)-[^18^F]FMeNER-D_2_, emission data were collected between 120 and 180 min post-injection—a time window validated by Arakawa et al. (2008)^17^ for producing reliable area under the curve (AUC)-based estimates of NET binding.

The AUC ratio from 120 to 180 min was calculated using the following equation:

AUC ratio = (^AUC^target / ^AUC^caudate)^−1^

This method closely aligned with binding potential values obtained from reference tissue models using full dynamic scans, supporting its applicability in clinical PET protocols. The mean injected radioactivity was 184.7 MBq (SD = 7.6), and the molar activity averaged 702.5 (SD = 237.2) GBq/μmol at the injection time. Data were reconstructed by filtered back-projection using a Hanning filter (6.3 mm, full-width at half maximum).

Magnetic resonance imaging (MRI) was obtained using a 3.0-T scanner (MAGNETOM Verio, Siemens, Germany). A 3-dimensional T1-weighted gradient echo sequence produced gapless thin sagittal sections (echo time = 1.95 ms, repetition time = 2300 ms, inversion time = 900 ms, flip angle = 9°, field of view = 250 mm, acquisition matrix = 256 × 256, slice thickness = 1 mm, and voxel size = 1 mm × 1 mm × 1 mm). No structural abnormalities were observed in the MRI results.

### Study Procedure and Treatment Administration

Baseline PET examinations were conducted for all 15 patients with MDD using (*S,S*)-[^18^F]FMeNER-D_2_. After the baseline examinations, patients were treated with duloxetine (20–60 mg daily), with the dose determined by the treating physician based on symptom severity. After 4–6 weeks of duloxetine administration, a second PET examination was performed. Plasma concentrations of duloxetine were measured immediately before and after the second examination using gas chromatography–mass spectrometry, with a lower limit of quantification of 0.1 ng/mL. Duloxetine administration was continued thereafter. Seven patients who showed symptom improvement over the course of time discontinued duloxetine by tapering over a period of 1–2 weeks in accordance with clinical guidelines, and underwent a third PET examination after a 2-month washout period. These successfully treated patients had received duloxetine for an average of 9.9 months before tapering. The remaining 8 patients who required a change in medication or additional treatment due to insufficient response to duloxetine were discontinued from study participation, and did not complete a medication washout or a third PET examination.

### Image Analysis

T1-weighted MRIs were coregistered with the corresponding PET images using SPM8 software (Wellcome Trust Centre for Neuroimaging, London, UK). Positron emission tomography images were spatially normalized to Montreal Neurological Institute (MNI) space (MNI 152 2 mm template) using the Diffeomorphic Anatomical Registration Through Exponentiated Lie Algebra (DARTEL) algorithm in SPM8.^18^ The Oxford thalamic connectivity atlas^19^ was applied to the spatially normalized PET images to extract time–activity curves for thalamic subregions, and regions of interest for the locus coeruleus and caudate were manually drawn based on established methods.^19^^,^^20^ The caudate was used as a reference region, as it is almost devoid of NET. The AUC ratios were calculated from 120 to 180 min post-injection.

Norepinephrine transporter occupancy was calculated using the following equation:


$$ \mathrm{Occupancy} \!\left( \%\right)\! =\!\frac{{\left(\mathrm{AUC}\, \mathrm{ratio}-1\right)}_{\mathrm{baseline}}-{\left(\mathrm{AUC}\, \mathrm{ratio}-1\right)}_{\mathrm{drug}}}{{\left(\mathrm{AUC}\ \mathrm{ratio}-1\right)}_{\mathrm{baseline}}} \times \! 100 $$

where (AUC ratio − 1)_baseline_ is (AUC ratio − 1) in the drug-free state, and (AUC ratio − 1)_drug_ is (AUC ratio − 1) after administration of duloxetine.

The relationship between the dose or plasma concentration of duloxetine and NET occupancy is described by the following equations:


$$ \mathrm{Occupancy}\ \left(\%\right)=\frac{D}{D+{\mathrm{ED}}_{50}}\times 100 $$

$$ \mathrm{Occupancy}\ \left(\%\right)=\frac{C}{C+{\mathrm{EC}}_{50}}\times 100 $$

where *D* and *C* are the dose and plasma concentrations of duloxetine, respectively. ED_50_ and EC_50_ are the dose and plasma concentrations inducing 50% NET occupancy, respectively. The classical Hill equation was used to describe the relationship between drug plasma concentration or dose and NET occupancy. GraphPad Prism version 6 was used to calculate ED_50_ and EC_50_.

### Test–Retest Reliability Analysis

Although (*S,S*)-[^18^F]FMeNER-D_2_ has been extensively used in clinical and occupancy studies to assess NET availability in the human brain, the test–retest reliability of (*S,S*)-[^18^F]FMeNER-D_2_ has not been systematically investigated. Therefore, we also evaluated the test–retest reliability of (*S,S*)-[^18^F]FMeNER-D_2_ using PET imaging in 5 healthy human participants, scanned twice under identical conditions (no drug) approximately 4 weeks apart. Details of the procedure are presented in the Supplementary Methods section of the Supplementary File.

### Statistical Analyses

Sample Size and Power Calculation: An a priori power analysis (G^*^Power 3.1.9.7) showed that data from 14 individuals would provide 80% power (α = 0.05, 2-tailed) to detect a large within-subject change in thalamic NET AUC ratio (Cohen’s *d* = 0.80). To allow for one dropout, we set a target of 15 participants. In total, 14 participants completed all primary assessments.

With 15 patients enrolled (7 completing all 3 PET sessions), our study had 80% power (α = 0.05) to detect a within-subject change of approximately Cohen’s *d* ≈ 0.8 or a correlation of *r* ≈ 0.7. Moderate or smaller effects may therefore have been missed. Notably, studies employing neuroreceptor occupancy PET^21^^,^^22^ usually include 5–20 participants because each subject undergoes multiple, resource-intensive scans. Therefore, our sample size fits within the accepted exploratory range. Two primary statistical analyses were performed in our study:

NET Occupancy Measurement: Differences in AUC ratio values between the first and second PET examinations in the 15 patients were assessed to calculate NET occupancy.

Paired *t*-Test Analysis: Differences in AUC ratio values between the first and third PET examinations were analyzed in the 7 patients who showed symptom improvement with duloxetine using a paired *t*-test. In addition, the correlations between the rate of change in AUC ratios and both post-treatment HAM-D scores and the percentage change in HAM-D scores were analyzed. Pearson correlation coefficients were used to evaluate these relationships.

All analyses were performed using SPSS, version 29 (IBM Corp., Armonk, NY, USA).

## RESULTS

### Patients

The demographic and clinical characteristics of patients at the pre-treatment, mid-treatment, and post-treatment stages are presented in Table 1.

**Table 1:** Demographic and clinical characteristics of patients at the pre-treatment, mid-treatment, and post-treatment stages

	Pre-treatment (*n* = 15)		Mid-treatment (*n* = 15)		Post-treatment (*n* = 7)	
	Mean	Standard deviation	Mean	Standard deviation	Mean	Standard deviation
**Age (years)**	39.7	10.3			47.0	6.4
**Sex (number of males)**	9		9		5	
**HAM-D scores**	21.3	4.8			3.7	1.2
**Duloxetine (mg)**			34	13.5		
**Treatment duration (months)^a^**					9.9	5.6
**Past episodes (number)**	1.3	0.46				
**Duration of this episode (months)**	4.6	3.9				

Abbreviation: HAM-D, Hamilton Depression Rating Scale.

a Treatment duration refers to the total time patients remained on duloxetine prior to discontinuation and the subsequent 2-month washout period. No other antidepressant medications were administered during the washout phase.

### Norepinephrine Transporter Occupancy

**Figure 1:**
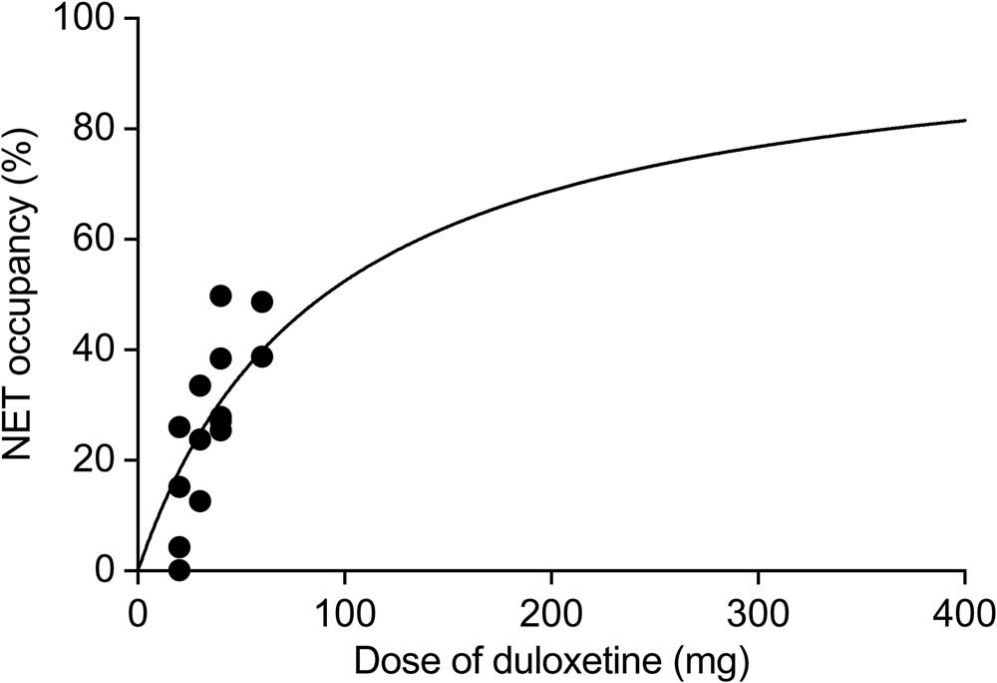
Norepinephrine transporter occupancy. Relationship between duloxetine dose and norepinephrine transporter (NET) occupancy is shown.

Norepinephrine transporter occupancy by duloxetine was calculated using data from 2 PET examinations with (*S,S*)-[^18^F]FMeNER-D_2_. The first examination was performed at baseline, before any medication, and the second examination was conducted 4–6 weeks after starting duloxetine treatment. The ED_50_ and EC_50_ values were calculated as 92.39 mg and 71.21 ng/mL, respectively, using GraphPad Prism version 6. The results are presented in Figure 1. Norepinephrine transporter occupancy by duloxetine was approximately 30%–40% across the doses studied.

### Comparison Before and After Treatment

The second part of the analysis focused on comparing NET availability in the brains of patients with MDD before and after duloxetine treatment. Positron emission tomography examinations were performed at 3 time points: at baseline, after 4–6 weeks of duloxetine administration, and a third examination for 7 patients who showed symptom improvement and completed the duloxetine treatment before tapering (mean duration, 9.9 months) and underwent a third PET examination after a 2-month washout period.

**Figure 2:**
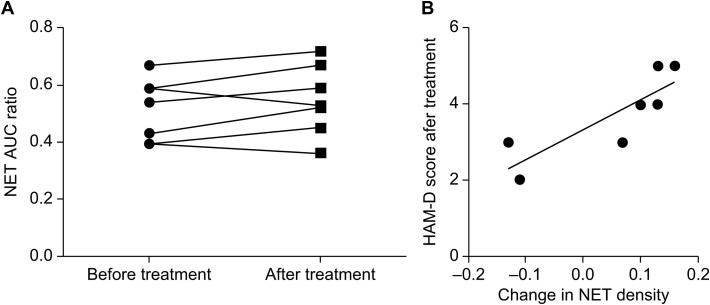
Comparison before and after treatment. (A) Norepinephrine transporter (NET) area under the curve (AUC) ratio at baseline (before treatment) and after 4–6 weeks of duloxetine treatment in patients with major depressive disorder. (B) Correlation between changes in NET availability and Hamilton Depression Rating Scale (HAM-D) scores after duloxetine treatment (*r* = 0.83, *P* = .018).

A paired *t*-test comparing NET availability before and after duloxetine treatment (baseline vs. post-treatment) showed no significant differences [raw AUC ratio values (AUC ratio – 1) for the thalamus (mean ± standard deviation), before treatment: 0.52 ± 0.10, after treatment: 0.55 ± 0.11], suggesting that duloxetine did not significantly alter NET availability during the treatment (Figure 2).

A significant positive correlation was observed between the change in NET availability and absolute post-treatment HAM-D scores (*r* = 0.83, *P* = .018). Here, ΔNET was defined as (AUC ratio − 1) baseline − (AUC ratio − 1) post; thus, larger ΔNET indicates a greater reduction in NET availability. A larger ΔNET (greater reduction in NET availability) was associated with lower post-treatment HAM-D scores. This aligns the interpretation with the correlation coefficient.

### Test–Retest Reliability Analysis

The test–retest reliability of (*S,S*)-[^18^F]FMeNER-D_2_ in PET imaging was evaluated using data from 5 healthy participants using Cronbach’s alpha, which yielded a high value of 0.973, indicating excellent consistency across repeated examinations. The intraclass correlation coefficient was also calculated, which showed a single-measure value of 0.770 (95% CI: −0.071 to 0.975) and an average value of 0.870 (95% CI: −0.154 to 0.987), both reflecting statistically significant reliability (*F*(4, 4) = 36.466, *P* = .002). These results confirm the reproducibility of (*S,S*)-[^18^F]FMeNER-D_2_ in PET imaging. The AUC ratios for each participant across the 2 test sessions are presented in Supplementary Figure S1.

## DISCUSSION

### Key Findings

This study aimed to evaluate the effect of duloxetine treatment on NET availability in the brains of patients with MDD using PET with (*S,S*)-[^18^F]FMeNER-D_2_. The key findings revealed that duloxetine led to approximately 30%–40% NET occupancy across doses ranging from 20 to 60 mg daily and that changes in NET availability were positively correlated with improvements in clinical symptoms, as measured by the HAM-D. To the best of our knowledge, this is the first study to monitor the long-term effects (over 6 months) of treatment in patients with a mental disorder using PET imaging without concurrent medication use. Our findings of test–retest reliability analysis also validate the use of (*S,S*)-[^18^F]FMeNER-D_2_ as a reliable tracer for NET measurements, offering the consistency needed for longitudinal studies or studies with small sample sizes, where stable measurements over time are crucial.

### NET Occupancy

Our findings align with previous research suggesting that clinical doses of duloxetine result in substantial NET occupancy in the brains of healthy participants.^23^ The calculated ED_50_ and EC_50_ values for duloxetine were 92.39 mg and 71.21 ng/mL, respectively, indicating that the drug effectively occupies the NET at therapeutic doses. This level of occupancy is consistent with findings from studies on other SNRIs and NET inhibitors, which have demonstrated similar occupancy rates in the treatment of MDD.^24–26^ Duloxetine has a markedly higher affinity for the SERT (Ki = 0.8 nM) than for the NET (Ki = 7.5 nM), resulting in greater SERT occupancy at therapeutic doses. Positron emission tomography studies indicate that 40–60 mg duloxetine achieves about 80% SERT occupancy, a level associated with efficacy in MDD. Although duloxetine’s NET occupancy (30%–40%) is lower than the 50% benchmark used for some drugs, its antidepressant efficacy in MDD likely stems from combined SERT and NET inhibition. Given that MDD involves both serotonin and norepinephrine systems, partial NET engagement together with strong SERT occupancy is thought to play a key role in its therapeutic effect. In a study conducted by our group,^27^ NET occupancy was measured from 2 PET examinations using (*S,S*)-[^18^F]FMeNER-D_2_ before and after a single oral dose of duloxetine (20, 40, and 60 mg). NET occupancy with duloxetine was 29.7%, 30.5%, and 40% at 20, 40, and 60 mg, respectively. The estimated duloxetine dose for 50% NET occupancy was 76.8 mg, with a corresponding plasma concentration of 58.0 ng/mL. We concluded that clinical doses of duloxetine achieve approximately 30%–40% NET occupancy in the human brain. These results suggest that 30%–40% NET occupancy by duloxetine is a key factor in its therapeutic effects. Our findings are supported by previous studies. Single-dose nortriptyline showed 16%–41% NET occupancy in healthy subjects depending on dose.^28^ Chronic nortriptyline (75–200 mg/day) resulted in 50%–70% NET occupancy in patients with depression.^29^ Milnacipran showed dose-dependent NET occupancy, increasing from 25% at 25 mg to approximately 50% at 200 mg/day.^25^ We have previously reported that single-dose duloxetine produces NET occupancy ranging from approximately 30% (20–40 mg) to 40% at 60 mg.^27^ In another study, quetiapine XR when administered for 6–8 days led to 19%–35% NET occupancy depending on dose.^30^ In patients with mood disorder, quetiapine XR also showed NET occupancy of 22%–34% at 2 weeks and up to 38% at 7 weeks.^31^ The observed NET occupancy aligns with duloxetine’s pharmacological action as an SNRI, enhancing the synaptic availability of both neurotransmitters, which is beneficial in alleviating depressive symptoms.

### Clinical Implications and Correlation with Symptom Improvement

The absence of NET binding changes after the 2-month washout, despite persistent symptom improvement, suggests that duloxetine’s antidepressant effects may not rely on sustained NET occupancy or downregulation. While acute NET inhibition may trigger symptomatic relief, long-term therapeutic effects may be maintained through other mechanisms, such as neuroadaptive changes or modulation of other neurotransmitter systems.

We also examined the relationship between NET occupancy by duloxetine and HAM-D scores at the 4- to 6-week time point, when both PET and clinical assessments were available. Although no significant correlation was found, this may reflect the relatively narrow range of observed NET occupancy (30%–40%) or limited statistical power. Nonetheless, these findings suggest that while NET occupancy is pharmacologically relevant, individual clinical response may depend on additional neurobiological factors beyond target engagement alone.

The positive correlation between change in NET availability and absolute HAM-D scores after treatment suggests that patients with greater reductions in NET availability exhibited fewer residual depressive symptoms. However, we did not find a significant correlation between changes in NET availability and changes in HAM-D scores, suggesting that the relationship may reflect a state-dependent association rather than a strictly proportional treatment response. One possible explanation for these observations is the variability of baseline HAM-D scores among participants. While the absolute post-treatment scores may directly reflect the clinical state after duloxetine treatment, percentage changes are influenced by the initial severity of depressive symptoms, potentially introducing heterogeneity into the relationship with NET availability changes. Another factor to consider is the sensitivity of HAM-D subscales to changes in specific depressive symptoms. Post-treatment scores may better capture the state of residual symptoms that align more closely with neurobiological changes. In contrast, percentage changes aggregate across all items, potentially diluting the relationship with NET-specific mechanisms. Future studies could address this issue by analyzing correlations between NET changes and alternative scales that more precisely reflect noradrenergic function.

These findings highlight the complexity of linking clinical outcomes with neurobiological markers and underscore the need to carefully select metrics when evaluating treatment efficacy. There is ongoing debate about whether HAM-D accurately reflects depression severity across diverse patient groups. Isacsson^32^ contends that, while the HAM-D may lack precision in cases of milder depression, it is effective for evaluating patients with HAM-D scores of 17 or higher, where antidepressant effects are more noticeable, and measurement reliability is enhanced. In our study, all participants began with HAM-D scores above this threshold, indicating that the scale was suitable for assessing depression severity and treatment response within our cohort. However, we did not include more granular symptom-specific scales, which could further clarify the impact of treatment on individual symptoms. Addressing this limitation in future research would offer a more refined understanding of therapeutic outcomes.

Our findings emphasize the complexity of MDD and its treatment, suggesting that while average changes in NET availability may not reach statistical significance across a population, individual responses can vary considerably and have important clinical implications. The positive correlation with HAM-D scores suggests that monitoring changes in NET availability could help predict treatment outcomes and personalize interventions for individual patients.

### Consistency with Previous Research

Studies have also shown that chronic treatment with SNRIs can reduce NET expression. For example, research on rats has demonstrated that prolonged administration of selective norepinephrine reuptake inhibitors, such as desipramine, reduces NET binding sites and norepinephrine uptake in brain regions like the hippocampus and cortex.^33^ These findings suggest that the decrease in NET availability observed in our study may contribute to duloxetine’s therapeutic mechanism and antidepressant effects.

### Comparison with Postmortem Studies

Our study provides essential in vivo evidence that aligns with and clarifies the mixed findings of previous postmortem research on NET alterations in MDD. Some postmortem studies indicate reduced NET binding in the locus coeruleus of patients with MDD,^4^ while others report variations in adrenergic receptor profiles, which may reflect complex changes in the norepinephrine system in MDD.^5–7^ Although our findings do not directly resolve inconsistencies in postmortem studies, they provide in vivo evidence of NET occupancy by duloxetine, offering a functional perspective that helps contextualize static postmortem observations and supports the clinical relevance of NET modulation in MDD. Furthermore, the significant positive correlation between changes in NET availability and HAM-D scores after treatment suggests that duloxetine’s NET occupancy may directly influence clinical outcomes, adding depth to postmortem findings and highlighting the therapeutic relevance of NET in MDD.

### Limitations

This study has several limitations. A major limitation of this study is its failure to account for the influence of other monoamines, such as serotonin, which also play a critical role in the pathophysiology of MDD. Duloxetine, an SNRI, affects both norepinephrine and serotonin reuptake, and changes in serotonin levels may have contributed to the observed clinical outcomes. Future research should focus on simultaneously measuring changes in both norepinephrine and SERTs to provide a more comprehensive understanding of the neurobiological effects of duloxetine and other SNRIs. Furthermore, the sustained antidepressant effects of duloxetine likely involve complex downstream neuronal adaptations and signaling pathways beyond immediate transporter occupancy. These deeper neurobiological changes should be explored for a comprehensive understanding of duloxetine’s therapeutic actions. In addition, duloxetine increases dopamine levels in the prefrontal cortex.^34^ This increase in dopamine occurs due to duloxetine’s inhibition of NET, which also has a high affinity for dopamine. As a result, by blocking NET, duloxetine indirectly raises dopamine levels. This effect is particularly significant in the prefrontal cortex, where dopamine transporters are scarce, and dopamine reuptake primarily relies on NET.^34^ Furthermore, the sample size was relatively small, and the dropout rate was high, with 8 patients needing changes in medication or additional treatments. This limited sized cohort and attrition may have consequently led to limited statistical precision; therefore, the present findings are preliminary and should be confirmed in larger, adequately powered studies. Future studies with larger sample sizes should also consider the differences between chronic and acute duloxetine treatment. In addition, the long-term effects of duloxetine on NET availability and its relationship with sustained clinical improvement require further exploration. It is also important to note that depression presents with a range of symptoms, and it is uncertain whether the HAM-D accurately captures those specifically targeted by SNRIs. More detailed symptom monitoring will be essential in future research.

Although no significant changes were observed in overall NET availability following duloxetine treatment, the positive correlation between changes in NET availability and improvements in HAM-D scores highlights a potential inconsistency that warrants further exploration. This discrepancy suggests the possibility of individual variations in NET-related mechanisms underlying symptom improvement. The observed association between NET availability change and HAM-D scores should be carefully interpreted, as HAM-D reflects residual symptom severity rather than a direct, comprehensive measure of clinical improvement. Future studies with larger sample sizes and more detailed longitudinal symptom assessments should further clarify and validate the relationship between NET modulation and treatment outcomes. Moreover, smaller changes in NET binding may have gone undetected due to small sample size, and our findings, particularly regarding the correlation, should be viewed as exploratory. A more detailed investigation into this relationship, potentially incorporating additional biomarkers or complementary imaging techniques, could provide deeper insights into the therapeutic effects of duloxetine. Moreover, the limited spatial resolution of PET images in small structures such as the locus coeruleus could lead to high variability in binding potential non-displaceable values, making subtle alterations difficult to detect in such regions.

Another limitation is the exclusive use of a single PET radioligand (*S,S*)-[^18^F]FMeNER-D_2_. While this radioligand is effective for measuring NET occupancy, incorporating other imaging techniques or biomarkers could provide a more holistic view of duloxetine’s neurobiological effects. Moreover, since duloxetine acts on both norepinephrine and serotonin, future studies should also assess SERT availability to better understand the combined effects of these monoamines in treating MDD. In addition, the European Medicines Agency recommends 60–120 mg/day of duloxetine for MDD. However, our study was conducted in Japan, where the Ministry of Health, Labour and Welfare approves treatment with duloxetine at a dose range of 20–60 mg/day and prohibits routine use > 60 mg/day. In terms of medical ethics, prescriptions exceeding the national maximum would have been off-label use and therefore were not considered in our protocol. Therefore, the generalizability of our findings in other parts of the world may be limited.

## CONCLUSION

In conclusion, this study demonstrated that duloxetine induces significant NET occupancy in the human brain, and that reductions in NET availability observed during treatment may be associated with post-treatment depression severity. While these findings support the involvement of norepinephrine transmission in MDD, the observed association between NET binding and HAM-D scores after treatment should be interpreted cautiously, as the latter reflects residual symptom severity rather than a direct measure of clinical improvement. Further studies with larger sample sizes and more granular longitudinal symptom assessments are needed to clarify the relationship between NET modulation and treatment outcomes.

Understanding changes in NET and their implications enhances our comprehension of the complex mechanisms behind antidepressants and may reveal new therapeutic targets for MDD and other neuropsychiatric disorders. In addition, future studies incorporating the role of other monoamines, such as serotonin, will provide a more comprehensive understanding of the neurochemical changes induced by SNRI treatment and their impact on clinical outcomes.

## Supplementary Material

Revised_Supplementary_Material_pyaf064

## Data Availability

The data that support the findings of this study were collected and analyzed within the institute and are not available openly. However, they are available from the corresponding author, S.M., upon reasonable request.
