# Supplementary material for: A longitudinal PET study on changes in brain norepinephrine transporter availability following duloxetine treatment in major depressive disorder
Source: Int J Neuropsychopharmacol. 2025 Sep 11;28(10):pyaf064. doi: 10.1093/ijnp/pyaf064 (PMC12489337; doi:10.1093/ijnp/pyaf064)
Supplement: Revised_Supplementary_Material_pyaf064 [file revised_supplementary_material_pyaf064.docx]

**A Longitudinal PET Study on Changes in Brain Norepinephrine Transporter Availability Following Duloxetine Treatment in Major Depressive Disorder**

**SUPPLEMENTARY MATERIAL**

**Supplementary Methods**

**Participants**

In this study, five healthy volunteers (mean age = 28 years, standard deviation (SD) = 8; two men, three women) underwent two separate PET examinations with the radioligand (*S,S*)-[^18^F]FMeNER-D_2_ each on different days to assess test-retest reliability.

**PET imaging procedure**

During each session, positron emission tomography (PET) data acquisition commenced 120 minutes following the intravenous injection of (*S,S*)-[^18^F]FMeNER-D_2_, with continuous imaging from 120 to 180 minutes. A Siemens ECAT EXACT HR+ PET scanner with a 15.5-cm axial field of view was used, and a head fixation device was employed to minimize motion artifacts. Attenuation correction was performed with a 10-minute transmission scan using a ^68^Ge-^68^Ga source, and data were reconstructed using a Hanning filter. The mean injected radioactivity was 189.9 MBq (SD = 13.6), and the molar activity averaged 566.44 (SD = 444.1) GBq/μmol at the injection time.

**Test-retest reliability analysis**

To evaluate the reliability of (*S,S*)-[^18^F]FMeNER-D_2_ PET imaging across repeated examinations, both Cronbach’s alpha and the intraclass correlation coefficient (ICC) were calculated using SPSS software, version 29 (IBM Corp., Armonk, NY, USA). Cronbach’s alpha evaluates internal consistency by examining the average correlation among measurements, with values above 0.7 indicating acceptable reliability and values above 0.9 signifying excellent reliability. Additionally, the ICC assesses the consistency between repeated measurements, with higher ICC values reflecting greater reliability.


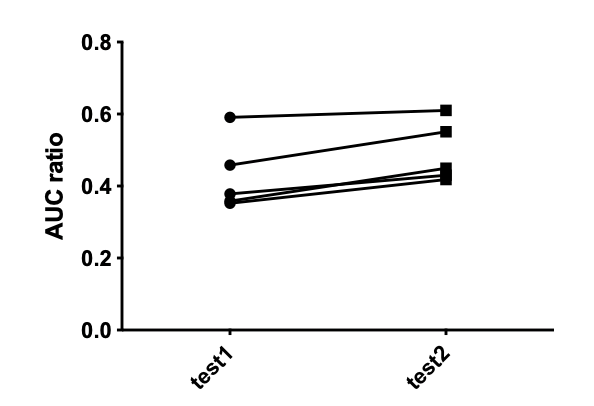


**Supplementary Figure S1.** Area under the curve (AUC) ratios for each participant across two test sessions (test1 and test2) show consistent AUC values with minimal variability between sessions, further corroborating the high reliability indicated by Cronbach’s alpha and ICC results.
